# Measuring Respiratory Motion for Supporting the Minimally Invasive Destruction of Liver Tumors

**DOI:** 10.3390/s22176446

**Published:** 2022-08-26

**Authors:** Dominik Spinczyk

**Affiliations:** Faculty of Biomedical Engineering, Silesian University of Technology, 40 Roosevelta, 41-800 Zabrze, Poland; dominik.spinczyk@polsl.pl

**Keywords:** destruction of liver tumors, measurement of respiratory motion, image-guided navigation, minimally invasive liver interventions

## Abstract

Objective: Destroying liver tumors is a challenge for contemporary interventional radiology. The aim of this work is to compare different techniques used for the measurement of respiratory motion, as this is the main hurdle to the effective implementation of this therapy. Methods: Laparoscopic stereoscopic reconstruction of point displacements on the surface of the liver, observation of breathing using external markers placed on the surface of the abdominal cavity, and methods for registration of the surface of the abdominal cavity during breathing were implemented and evaluated. Results: The following accuracies were obtained: above 4 mm and 0.5 mm, and below 8 mm for laparoscopic, skin markers, and skin surface registration methods, respectively. Conclusions: The clinical techniques and accompanying imaging modalities employed to destroy liver tumors, as well as the advantages and limitations of the proposed methods, are presented. Further directions for their development are also indicated.

## 1. Introduction

Liver tumors are a serious health concern, and are classed as benign or malignant [1]. Primary liver cancer is the sixth-most frequent cancer and the fourth-leading cause of death from cancer globally [2]. In 2018, it occurred in 841,000 people and resulted in 782,000 deaths globally [2]. The most effective method of treating metastatic lesions in the liver is their resection with a healthy tissue margin, whereby the following conditions must be met: no extrahepatic metastases; localization of the lesion in one lobe without invasion of the liver cavity vessels; and maintaining a 1 cm margin [1]. In this regard, there are several minimally invasive treatment techniques available, including radioembolization, transcatheter arterial chemoembolization, and various types of ablation. These techniques are commonly used for non-resectable lesions, i.e., those that cannot be completely removed [1,2,3].

Percutaneous ablation of pathological changes in the liver is a challenge for contemporary interventional radiology, which is mainly due to the size and architecture of the organ. Furthermore, different types of pathological change in the liver are not clearly visible with the imaging modalities available, which include magnetic resonance imaging (MR), computed tomography (CT), and ultrasound (US), without the use of contrast, making their use difficult during procedures [4,5,6].

The main technology that responds to these challenges are image navigation systems that allow the working tip of the operating tool to be guided to the location of the lesion [6]. Classically, image navigation systems have used preoperatively acquired layered images to create a personalized model of the patient’s anatomy through the use of position tracking systems, which also determines the position of the surgical tool during the procedure [6]. Such an approach is sufficient for the treatment of rigid organs. However, navigation systems for parenchymal organs, which incorporate intraoperative images alongside the preoperative model of the patient’s anatomy, are still in development. Use of this approach will allow for increased accuracy of image navigation systems by visualizing local deformities during the procedure, in real time [7].

Techniques for modeling breathing movements can be categorized by the degree of invasiveness of the surgical techniques they are used alongside. Minimally invasive techniques include stereoscopic laparoscopic reconstruction of point displacements on the surface of the liver. Meanwhile, non-invasive techniques include observation of breathing based on external markers placed on the surface of the abdominal cavity and recording of the abdominal surface during breathing. Selected studies on the most common invasive and non-invasive methods of respiratory movement modeling are discussed below.

Measurement of respiratory movements is also used in invasive methods that utilize laparoscopic images, and non-invasive methods that record the displacement of skin markers during the procedure. The aim of this study is to compare the invasive and non-invasive techniques used for the measurement of respiratory movements. The article discusses the theoretical basis, the proposed experiments, the results obtained, and the possibility of using these techniques to support the minimally invasive destruction of focal lesions in the liver.

## 2. Materials and Methods

### 2.1. Minimally Invasive Methods of Measuring Respiratory Movements of the Liver

#### Laparoscopic Stereoscopic Reconstruction of Point Displacements on the Surface of the Liver

In current state-of-the-art technology, a classic laparoscopic camera is monocular and closed in a rigid housing. There are depth reconstruction algorithms for single-monocular cameras, but due to the lack of characteristic points and the wrapping of the liver surface during respiratory movement, these techniques are very difficult to implement. This solution to this is general in nature as it works for any patient for any section of the liver surface observed by the laparoscopic camera through the trocar being placed in different positions.

A more common and proven approach is the use of stereovision algorithms for depth reconstruction, which are based on how the human visual system reconstructs depth [8]. However, the disadvantage of this approach is the need for a laparoscopic stereo camera. Spinczyk et al. proposed a method based on a system of two calibrated laparoscopic cameras, consisting of the following stages: calibration of the laparoscopic cameras, finding points of correspondence in the images from the left and right cameras, and spatial reconstruction of the points using the triangulation algorithm in the sequence of images from the recording of the procedure [9]. The authors achieved this using point camera models and the Tsai camera calibration algorithm [10]. Correspondence of the points was determined manually and the reconstruction of the position of the point in three-dimensional space was found by solving the problem of correspondence on the basis of two monocular cameras [8]:

(1) $$X=\tau \left(P_{L},\;P_{P},x_{L},\;x_{P\;}\right)$$

where: τ represents the triangulation algorithm, $P_{L},\;P_{P\;}\;$represents projection matrices for left and right monocular cameras, $x_{L},\;x_{P\;}$ represents the coordinates of the points corresponding to the left and right cameras in the image, and *X* represents the position of the point of the world coordinate system (3D).

The triangulation algorithm described above was used to reconstruct the spatial position of the points on the liver surface, observed in the content of images from monocular laparoscopic cameras. Solving the problem of reconstructing the depth of the observed scene on the basis of images of two monocular cameras required the determination of two types of correspondence:

- the relevance of the images in the video sequences of the left and right cameras—in the time domain—is described below,
- the correspondence of points in the images of the left and right cameras—in the spatial domain—was determined manually on the basis of finding local maxima in the image content.

Synchronous triggering of cameras yields the best solutions for timing. Standard laparoscopic video tracks do not have such mechanisms built in, so the research used a time-stamped approach. Each video frame received from both cameras was timestamped. In order to receive the frames evenly, the sequence from individual video cameras was received with the use of synchronized streams with the use of a frame grabber hardware card. The timestamp value was determined from the global counter value, which remains the shared resource available to said threads responsible for receiving video sequences. At the reconstruction stage, it was allowed to reconstruct on the basis of a pair of frames from both cameras, the maximum shift of the time stamps of which did not exceed the time resolution of the video sequence. If the timestamp offset did exceed this value, such frames were discarded.

Optical properties of the surface of the liver were determined by the random sampling consensus (RANSAC) [11], and the Lucas–Kanade method [12], for finding the correspondence of points.

### 2.2. Non-Invasive Methods of Measuring Respiratory Movements on the Patient’s Surface

The most commonly used technique is the commercially available video metric real-time patient position management system, which allows for planning of the patient’s position during radiotherapy. Schaerer et al., used an optical tracking system to track the surface of the abdomen using AlignRT, which uses the triangulation algorithm presented above. The problem of finding the spatial relevance of points was solved by projecting a stereovision pattern onto the abdominal surface using a projector integrated into the camera housing. Depending on the size of the region of interest, the system works with a time resolution in the range of 1–15 frames per second (fps). The mean error of matching the markers for the consecutive surfaces in the time sequence was 1.61 mm [13].

#### 2.2.1. Observation of Breathing Based on External Markers Placed on the Surface of the Abdominal Cavity

**Figure 1:**
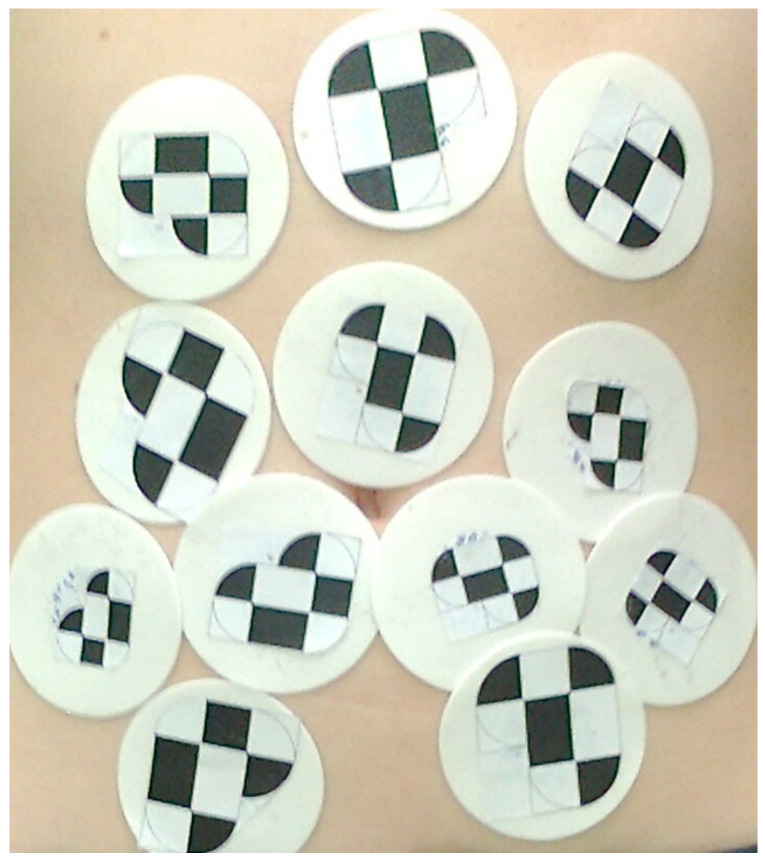
Diagram of the position of skin markers for respiratory motion measurement.

The proposed approach uses the Claron Hx40 video metric tracking system from ClaronNav, Toronto, Canada [14] to identify target objects and tracked them based on Xpoints, which are places of intersection on a flat surface of black and white areas. The distance between two points of intersection is defined by the vector, and two vectors of a rigid configuration and different length, meeting the condition of the angle between them within 8–172 degrees, define the simplest marker. Each marker should be unique in size so that it can be distinguished from other markers. Using these assumptions, markers were proposed, and their distribution scheme is shown in Figure 1.

Use of a rigid stereo camera, calibrated and triggered synchronously, eliminated the problem of finding the time correspondence in the sequence of images from the left and right monocular cameras, while the design of distinguishable markers unequivocally solved the problem of finding the correspondence of points. Spatial reconstruction of points by the Claron system uses the triangulation algorithm described above. In contrast to the studies carried out for the reconstruction of the points of the liver surface, where the stereo camera was mounted from two laparoscopic cameras, the measurements shown were made with a stereo Claron Hx 40 camera, calibrated at the factory. According to the manufacturer’s data, the root-mean-square of calibration error is 0.2 mm, and the spatial reconstruction error component during the movement of markers is 0.14 mm. The camera worked at a resolution of 1024 by 768 points, and at a rate of 5.4 fps for the twelve-marker configuration. Before taking the appropriate measurements, preparation of the stand required a series of tests to determine the size of the markers, the position of the patient, and the lighting conditions, so that the camera could capture all of the markers. A number of tests were then carried out to observe the respiratory process. Shallow breathing was observed for 60 s, then deep breathing was observed for the next 60 s. The tests were carried out on 12 volunteers, 6 male and 6 female.

#### 2.2.2. Registration of the Surface of the Abdominal Cavity during Breathing

Collecting the data directly from the abdominal surface during breathing meant it was possible to record and model the respiratory movements of the surface. We used the most widespread surface fitting algorithms is the iterative closest point (ICP) algorithm, which has been independently presented by several authors [15]. The algorithm treats the surface as a set of points and if the surface is represented differently, it is converted to a point cloud though a sampling of the surface. The result of the algorithm’s operation is finding the searched transform, after which the source file is matched with the target file.

ICP is an iterative algorithm, where each iteration consists of two steps. In the first step, the correspondences of points in the matched source and target sets are found, where the criterion of finding matches is the Euclidean distance. In the second step, a new version of the resulting transform is computed, and both sets of points are placed on a common geometrical frame of reference. For each point in the source file, the entire target set is searched, and the closest point is chosen as its counterpart. The pairs of equivalents determined in this way are substituted into the Equation (2):

(2) $$f\left(R,\;T\right)=\frac{1}{N_{s}}\;\overset{N_{s}}{\underset{i=1}{\sum }}\|R_{i}-Rot\left(T_{i}\right)+Trans\left(T_{i}\right){\|}^{2}$$

where: *R* represents the target set (stationary), *T* represents the source set (matched), *Rot* and *Trans* represent components of the resulting transformation, and *N* represents the number of points in the point cloud. The final transform resulting from the operation of the algorithm is a combination of transforms from individual iterations. The calculations are performed by minimizing the mean square measure of error between two sets of points, by means of an analytical solution of the least squares problem [15]. One of the tests that was carried out was an attempt to record the abdominal surface and thorax for the two opposite breathing phases using the classical ICP algorithm. A multi-threshold Otsu filter [16] was used to find the patient’s surface on computed tomography images in both phases of the respiratory cycle after a prior smoothing of the image with a Gaussian low-pass filter, with which the patient’s body was segmented. Then, after pouring holes in the surface of the body representing the patient, the contour was found using a gradient filter.

Disadvantages of the classic ICP algorithm have been described in the literature [15]. Indeed, the algorithm’s convergence depends on how close to the global optimum the initial value of the transform lies, which is a parameter of the algorithm. Furthermore, finding equivalents is a complex operation, as for each point in the source set, an equivalent is searched for in the entire target set. Meanwhile, optimization of the quadratic measure is sensitive to outliers, assuming a uniform noise distribution with a normal distribution value that is expected to be equal to zero. In connection with the above, the algorithm may be modified in a number of distinct ways [15]. When selecting subsets of matched points, matched sets may require size limitation, which significantly affects the algorithm execution time. Additionally, finding equivalents affects the correctness of transform changes in successive iterations, which determines the quality of equivalents, and can be achieved through quantitatively differentiating the degree of relevance, rejecting incorrect equivalents, and applying a method of minimizing the error metric. Based on the above-mentioned criteria and our own experimental research of the classical algorithm, modifications to the method of searching for equivalents and finding the resulting transformation were proposed.

##### Proposed Changes in the Method for Searching for Equivalents

The following matchmaking changes were made:

- Representation of the point cloud by means of a flat table was abandoned in favor of a tree structure.
- The Euclidean distance condition was replaced by searching for an equivalent along the normal vector of the destination point.
- The search for the best match was preceded by initially selecting a starting set with the condition of the maximum difference of angles between the normal vectors of potential equivalents.

##### Proposed Changes in the Method for Finding the Result Transform

Based on Amberg’s proposal, an independent affine transform was introduced for each point of the cloud. This approach can be called a generalization of the classical ICP algorithm, where for each point of the input set, an independent transform is found as a result of calculations. Therefore, it can be said that each of the points is treated as a source subset, and the equation of the similarity measure contains three components (3) [17]:

(3) $$E\left(X\right)=E_{d}\left(X\right)+\alpha E_{s}\left(X\right)+\beta E_{l}\left(X\right)$$

where:

*Ed* (*X*) is a measure of the distance between the points of the target set and the transposed points of the source set, with each point of the target set having its own transformation.
*Es* (*X*) is a measure of point cloud stiffness that uses the cloud topology matrix and is created on the basis of neighborhood of points, which prevents the cloud shape from changing during algorithm iteration. The alpha coefficient represents the stiffness coefficient, which is changed in the course of the algorithm’s operation at moments when the difference of transforms in subsequent iterations is smaller than the given threshold, and this represents the situation when the source set stops approaching the target set.
*E_l_*(*X*) is the term that introduces correct pairs of equivalents on the basis of known marker positions in the input and target set, whilst the beta ratio represents a measure of the quality of matching.
*α* is a stiffness vector influencing the flexibility of the point cloud. The implementation of the process of finding a new transform of the non-rigid version of the ICP algorithm consists of two loops. In the outer loop, the stiffness coefficient is reduced to allow for ever greater local deformations of the initial shape in subsequent iterations. Then, for a given value of the stiffness coefficient, new values of the coefficients of the resultant transformation in the inner loop are calculated.
*β* is a weighting factor that enables the gradation of the influence of individual reference points.

Evaluation of the proposed methods of finding equivalents and transforms for individual points of the input set was carried out on data generated from the abdominal surface points during the inspiration and exhalation phases of the respiratory cycle, which was obtained from the time-of-flight SR4000 camera using MESA Imaging, Swiss Laser Net, Wollerau, Switzerland [18]. Based on the idea proposed by Amberg, an independent implementation of the non-rigid version of the Nearest Iterative Point algorithm was made. The four-neighborhood configuration was applied in the topology matrix, whilst the k-tree structure was adopted as the internal representation of the point cloud, with 1 cm squares glued to the abdominal surface used as markers. Finding equivalents was carried out using the following criteria: Euclidean distance of a point, which is the classical approach, and a modified version of the Horn algorithm, using the normal surface with the initial rigid registration [19]. To assess the quality of the registration, the global distance measured on the source and target sets was used. This was defined as the distance of the points transformed by the resultant transform of the input cloud to the nearest point of the target cloud, which were calculated from the set of points of the input cloud.

A quality assessment process was also carried out to find matches. The correctness of the equivalents is known in advance for rigid clouds based on the algorithm of their generation. For respiratory clouds based on the position of skin markers, the closest cloud points are in the source and target point clouds. The cloud points found in this way constitute model pairs of equivalents. Then, the actual pairs of equivalents are compared with the reference pairs, which defines the average error of matching the equivalents for all markers. Due to the lack of universal local measures of matching for the analysis of the matching process, a proprietary measure called the “match map” was proposed, which represents the number of points it attracted in the matching process for each point of the target cloud.

## 3. Results

### 3.1. Stereoscopic Reconstruction of Point Displacements on the Surface of the Liver

Accuracy of the measurements was validated using a phantom, with a mean error of 3.9–4.2 mm found. The following phenomena were described: surface curl, where points on the surface appeared and disappeared in a time sequence; light reflections, with changes in the distribution of image element values dependent on the lighting conditions that changed with the location of the liver; and shape deformations, with significant surface deformations making it difficult to determine the constant region of interest in the sequence time that significantly impeded the use of known tracking and point location approaches.

### 3.2. Observation of Breathing Based on External Markers Placed on the Surface of the Abdominal Cavity

**Figure 2:**
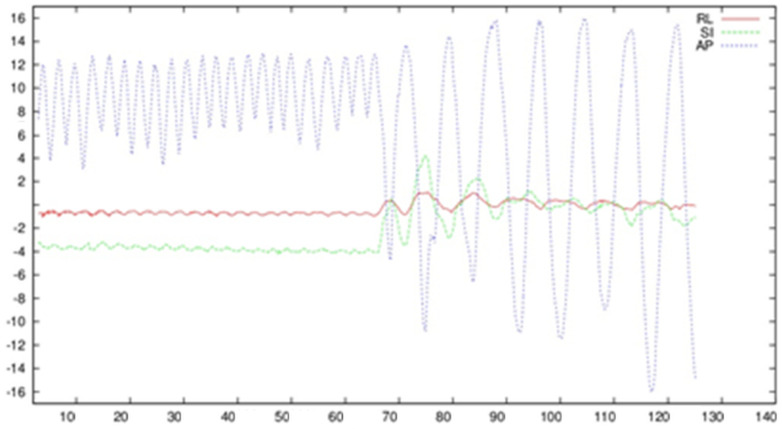
Timing diagram showing marker movement on the X−axis [mm] of the skin marker located closest to the naval, and respiratory motion in time on the Y−axis [s]. Motion was measured in typical projections: anterior−posterior (AP) represented by the blue color; superior–inferior (SI) represented by the green color, and right−left (RL) represented by the red color.

Exemplar plots representing displacement of the central marker, which was located closest to the navel, are presented in Figure 2. This confirmed the possibility of tracking respiratory movement with the use of skin markers and demonstrated the dependence of respiratory movement on individual characteristics.

In general, the largest component of movement was recorded in the anterior–posterior plane (Figure 2), which is a typical situation. Furthermore, the magnitude of movement was greater during the period of deep breathing, with unique marker deflection observed.

### 3.3. Recording of the Surface of the Abdominal Cavity during Breathing

**Figure 3:**
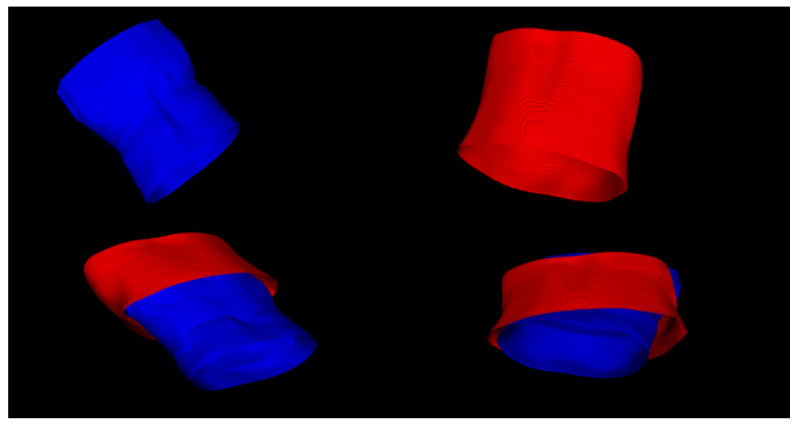
Example of the surface of the chest and abdomen for exhale (top left) and inhale (top right). The surfaces in the surface registration process before (bottom left) and after rigid registration (bottom right).

Figure 3 shows exemplar results of segmentation and matching of the skin surface, with a significant approximation of the surface observed. However, it was not possible to completely adjust the surface in the respiratory phases on the basis of a single global rigid transform proposed by the classical ICP algorithm, due to the change in the shape of the surface during the respiratory process.

**Figure 4:**
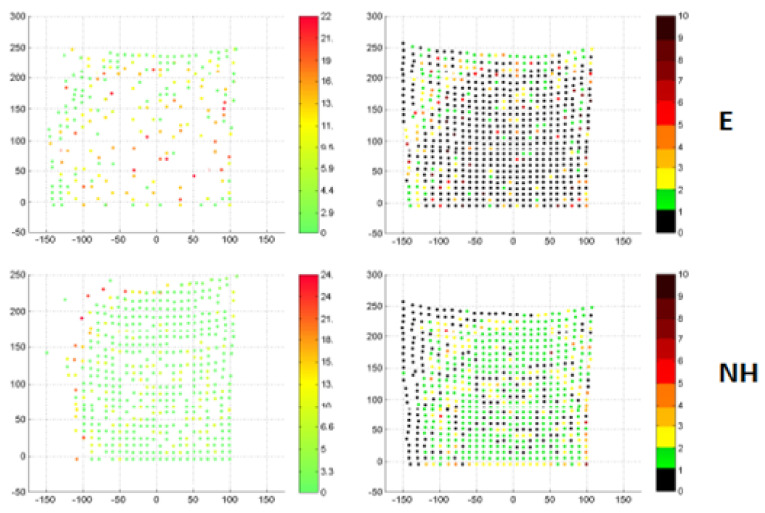
Example of the results of the non−rigid registration of the abdomen surface of the example person in the opposite breathing phase. The surface corresponding to the exhale phase is presented in every picture, as target surface. Distance map [mm] of the 95th percentile (left column) and correspondence map [number of units] (right column) for two modifications of the iterative closest point algorithm finding correspondence: Euclidean distance (top row, *E*), normal shooting with initial rigid registration (bottom row, *NH*) [The color of point in the distance map represents the distance to the nearest point in the registered source surface. The color of point in the correspondence map represents the number of correspondences of this point found in the source surface].

When analyzing the distance maps (left column) and correspondent maps (right column) in Figure 4, the correctness of the non-rigid cloud fit at the level of the average Euclidean distance below 8 mm can be observed. Analyses of the distance maps demonstrate that there are missing points, in particular for finding equivalents based on the Euclidean distance. Such “missing points” were due to a local phenomenon of attracting many points of the source cloud by a single target point, which caused the input cloud to converge locally to the point. A global measure of this is the size of the target cloud point group, attracting only one point in the input cloud.

In order to minimize this phenomenon, the proposed modification consisted of looking for equivalents along the normal vectors of the source points. The mere introduction of normal vectors resulted in a deterioration of the results in relation to the Euclidean distance, which was used as a reference point. Additionally, the use of rigid registration and the search for correspondents along normal vectors, using the known coordinates of markers in the source and target set, was applied. Indeed, increasing the group of target cloud points attracting single points of the source cloud was a necessary condition for improving the quality of the matching process.

## 4. Discussion

In the treatment of liver tumors, the preoperative model of the patient’s anatomy does not determine the choice of the technique for performing the procedure, with several options available for the destruction of focal liver lesions:

- Percutaneous ablation under the control of CT/external ultrasound probe.
- Ablation using the laparoscopic technique, which uses a laparoscopic trocar under the control of an image from a laparoscopic camera/ultrasound probe.
- Open surgery under the control of an intraoperative probe.

Standard radiological diagnostics describe liver lesions by specifying the location in the form of a segment number and relative position within the segment, as well as its approximate diameter [1,5]. With this information and the pre-operative patient anatomy model at hand, the operator can pre-plan the entry point and the trajectory of the tool. However, successful completion of the planning phase does not guarantee that the procedure will be performed according to the indications of this phase. Indeed, difficulty in locating the lesion in operating room conditions may result in a choice of different intervention techniques through cutaneous, laparoscopic, or even open surgery which ultimately affects the choice of respiratory movement measurement technique.

During open surgery, the respiratory movements of the liver lesion can be followed by an ultrasound intraoperative transducer. Due to the fact that the probe is applied directly to the organ wall, the ultrasound penetrates to smaller depths, which allows the use of higher frequencies and results in better image quality. The technique of image navigation is also used in open surgery. The suggested navigation technique incorporates a preoperative 3D liver model based on diagnostic 4D MRI scans and intraoperative contrast—via cone-bean computed tomography imaging and electromagnetic tracking (EM)—of the liver surface. The system’s average accuracy was 4.0 ± 3.0 mm [20].

Using the laparoscopic technique introduces certain conditions, and as a result of insufflation, it is not possible to observe the abdominal cavity through a cutaneous ultrasound probe. The available imaging tools include a laparoscopic head and a laparoscopic camera. However, due to its specific design, the laparoscopic probe has a limited field of view and requires the operator’s skill in positioning it. Additionally, due to the flexibility of the laparoscopic ultrasound (LUS) and the lack of optical visibility, the use of an electromagnetic position tracking system is required [21]. Other researchers reported that a prototype of a combined, EM-tracked laparoscope and LUS system using representative calibration methods showed a root-mean-square point localization error of 3.0 mm for the laparoscope and 1.3 mm for the laparoscopic ultrasound probe [22,23]. Nonetheless, with a laparoscopic stereoscopic camera, the problem of depth reconstruction is significantly simplified, as is tracking of selected points in a stereoscopic image. However, using a narrow-baseline stereo laparoscope may increase triangulation errors [23]. Laparoscopic deformations were significantly larger than deformations associated with open surgery. Using methods of deformation correction based on the registration of non-rigid images or on biomechanical models of organ tissue, it is possible to obtain target error of 6.7 ± 1.3  mm and surface error of 0.8 ± 0.4  mm [24].

During percutaneous intervention, the position of skin markers can be tracked, and the patient’s surface data can be recorded during the procedure. By analyzing the temporal plot of the central marker displacement shown in Figure 2, unique marker deflection during deep breathing as a function of time was observed. In the context of imaging navigation systems for the destruction of liver neoplastic lesions, this indicates the need to stabilize breathing at the time of insertion of the ablation needle in order to precisely determine the phase of the respiratory cycle. Thus, it can be synchronized with the phase for which the patient’s anatomical model was generated and the repeatability of the patient’s breathing depth. This is further supported by the use of general anesthesia during the ablation process, which results in greater control over the patient’s respiratory process. High-frequency jet ventilation can also be used to minimize respiratory movements, though this is limited by the availability of the apparatus and general condition of the patients health [25].

An important element of a position tracking system is the estimation of errors. In clinical practice, there are three types of optical tracking systems: video metric tracking, tracking systems operating in the infrared band, and electromagnetic systems. Optical systems require a line of sight between the camera and the markers and, in both classes of optical systems, a decrease in accuracy in the direction of the system’s viewing axis as it moves away is observed. Video metric systems are more sensitive to lighting conditions and the speed of movement of the tracked markers in relation to systems operating in the infrared band [26]. Meanwhile, electromagnetic systems are characterized by a smaller working volume, are less accurate than optical systems, and are sensitive to disturbances caused by electric and magnetic fields, caused by other technical equipment, among other factors. Furthermore, they usually require active markers, which requires power cables to be connected to the markers. However, their main advantage is that there is no need for optical visibility between the field generator and markers.

Tracking the respiratory movements of the patient’s surface with the use of time-of-flight cameras does not result in a very accurate measurement of single points. However, it presents an additional opportunity to observe local deformations of the surface that can be used when creating models of respiratory movements. It is also possible to increase the accuracy of matching individual surface fragments by taking into account the uncertainty of the acquisition of the location of individual points on the surface and the anisotropic nature of data noise [27]. Surface models are also extended to volumetric models, where the patient’s body is represented in the form of a tetrahedron mesh, which allows the measurement of volume changes during the respiratory cycle [28].

Reconstruction of the movements of anatomical structures is the subject of much research, when Fourier transform, vector auto-regression modelling, extended Kalman filter, and tracking laparoscopic instruments are used by marking them with colored markers.

Limitations of the methods described in the context of deformation modeling also include other sources in addition to breathing movements. Changes in the patient’s physiology, displacement of the intestines, change in weight, the degree of stomach filling and the impact of other diseases should be accounted for. Regardless of the technique used for modeling the breathing movements, the operator’s perception of the depth of the operating field remains a challenge. In order to overcome this barrier, attempts are being made to implement augmented and mixed reality systems into clinical practice. Using a head mounted display [29] allows for the presentation of the observed objects as a three-dimensional hologram. This improves depth perception and allows easier orientation between observed objects [30]. However, such systems are currently at the research stage [31,32].

## 5. Conclusions

Breathing motion is an important factor which influences the position of the nodule during therapy. Difficulty in locating the lesion in operating room conditions may result in a choice of different techniques to measure it. Therefore, different techniques of measuring breathing motion were presented. The advantages and disadvantages of which, as well as the achievable accuracy, were presented in the article.
